# Planar, wide-band omnidirectional retroreflector using metal-only transmitarray structure for TE and TM polarizations

**DOI:** 10.1038/s41598-022-15540-9

**Published:** 2022-07-04

**Authors:** Ali Pesarakloo, Mohammad Khalaj-amirhosseini

**Affiliations:** Electromagnetic Waves Propagation Laboratory, School of Electrical Engineering, Iran University of Science and Technology, Tehran, 1684613114 Iran

**Keywords:** Electrical and electronic engineering, Applied physics

## Abstract

In this paper, a novel planar and wideband metal-only retroreflector is proposed that efficiently reflects the obliquely incident electromagnetic wave along its incident direction in omnidirectional angle range. The means of omnidirectional is the capability of retroreflectivity in all azimuth angles $(\varphi_{i} )$ and in a wide elevation angle $(\theta_{i} )$ range. The proposed structure consists of a symmetrical transmitarray structure with beam scanning capability in which a metal plate is placed instead of the feed. The transmitarray is designed by using the generalized multifocal approach in which the beam scanning capability is possible via feed displacement and the phase of the elements has azimuthal symmetry that causes omnidirectional property. Now by placing a metal plate at the location of feed displacement and provided that the focused waves are nearly perpendicular to the metal plate, the plane wave is reflected back along its incident direction i.e. the retroreflectivity property is obtained. The proposed retroreflector simulated by CST STUDIO software and fabricated with laser cut technology. The results of measurement show an omnidirectional retroreflectivity with half-power (3-dB RCS level) elevation field of view of 60° (− 30° to 30°) in the frequency range of 8.5–10 GHz (approximately 17%) for both TE and TM polarization.

## Introduction

A retroreflector is a device that reflects light along its incident direction over a continuous range of incident angles^1^. The retroreflector can be used in two major fields; civil and military. In civil field, this can be used for navigation safety (aviation and marine), RF identification (RFID), satellite communication, and automotive collision avoidance. The mechanism of navigation safety is that in response to a signal from a friendly interrogating unit, it is desirable that the reflected wave is scattered monostatically back to the interrogator at a specified frequency corresponding to the target. Since the target is moving, the angular direction of the signal entering the target is variable, and so the retroreflector structure must operate in a wide angular range and at a certain frequency. RFID refers to a wireless system comprised of two components: tags and readers. The reader is a device that has one or more antennas that emits electromagnetic interrogation signals and receives identifying signals back from the tags attached to objects. Thus the tag must be a retroreflector structure and since tagged objects can be placed in different positions relative to the reader, the tag structure needs to be a retroreflector with a wide angular range. In addition, since the identifying signals are different digital data, the tag needs to have wide frequency bandwidth as well. In satellite communication, retroreflector structures are placed on satellites and used as a transponder, similar to navigation safety applications. In automotive collision avoidance systems, a signal is sent from one automotive to the surroundings, and if there are other automobiles around it, it is desirable that the reflected wave from the surrounding automobiles is scattered monostatically back to the main automotive. In this application also since the automobiles are moving, the angular direction of the signal entering the surrounding automobiles is variable, and so the retroreflector structure must operate in a wide angular range and at a certain frequency.

In military field, the retroreflector structures can be used for stealth and deception applications. In the field of stealth applications, sometimes there are small vehicles that have a small RCS and it is desirable that it can be detected by friendly radars but not by hostile radars. In these cases, it is desirable to use frequency-limited retroreflector structures mounted on small vehicles. Then the target will be detectable within the frequency range of a friendly radar while at other frequencies appears invisible. In this case, also, since the small vehicle can be moving, the radiated electromagnetic wave by the radar impinges on the target from different angles, and therefore requires a retroreflector structure with wide angular range and at a certain frequency. In deception applications, high RCS fake objects are required to be easily detected as targets by hostile radars. These fake objects are called decoys. Because the radiated wave by hostile radars can impinge on the decoys from different angular directions, in this case also retroreflector structures with wide angular range are needed.

In these applications, the general characteristics which are preferred for the retroreflector would be the ability for a wide-angle of incidence, the handling of both TE- and TM-polarized waves, high efficiency, low-profile, lightweight, low loss, low cost, and fabrication simplicity^2^. In addition, in some applications (such as RFID) a wide frequency bandwidth is also needed.

To the best of our knowledge, the first structure with retroreflectivity property was cat’s eye in the optics domain that was invented by Murray^3^ in 1927 and was used in advertising signs and then road marking^4^. The invented structure comprised of a plano-convex lens and a concave reflector arranged behind the plane rear surface of the lens. The mechanism is that the convex lens converges the incident beam onto the concave reflector and the reflector bounces the beam back along the incident direction. The first detailed study for cat’s eye retroreflectors was done by Beer and Marjaniemi in 1966, which was about wavefront error and construction tolerances^5^. Then the first analysis of the cat’s eye retroreflector was done by Snyder in 1975 using the paraxial ray matrix approach^6^. Finally, in 2017, Arbabi et al. proposed^1^ a planar near-infrared cat’s eye retroreflector composed of two layers of silicon nanoposts with a normal incidence efficiency of 78% and a large half-power field of view of 60° (± 30°).

Historically, the next retroreflector structure is corner reflector that was proposed by Spencer and Duboc^7^ in 1943. A corner reflector consisting of two or three electrically conductive surfaces which are mounted crosswise (at an angle of exactly 90 degrees) and causes the incoming electromagnetic waves are backscattered by multiple reflections accurately in that direction from which they come. Theoretical and experimental works show that the corner reflector provides half-power retroreflection in the range of ± 20°^8,9^. The main disadvantage of the corner reflector is that the structure is large and takes up a lot of space.

With the advent and development of dielectric lenses such as Luneburg lens introduced by Luneburg^10^ in 1944, a retroreflector structure using the Luneburg lens was invited by Kelleher^11^ in 1958 in which a Luneburg lens is partially covered by a metalized cap. This structure can facilitate half-power retroreflection across a wide angular range of about ± 50°^9^. However, it is limited by its large size, heavy weight, and relatively expensive fabrication.

The next retroreflector structure is Van Atta array that was invented in 1959 by Van Atta^12^. It consists of pairs of antenna elements equally spaced from the array center with equal-length or multiple wavelength difference transmission lines. The arrangement of the array causes a reversal of this phase progression for the outgoing signal, causing it to retro-reflect back in the same direction. With the advent of microstrips and striplines, the planar Van Atta array retroreflectors also were established that a wide angular bandwidth of over ± 30° half-power retroreflection has also been demonstrated^13^. However, the Van Atta structure has a few drawbacks, such as the transmission line network used has losses, which degrade the efficiency of the overall structure; and the transmission line network structure becomes very complex as the array size increases. This makes Van Atta array impractical for aperture lengths of several wavelengths and beyond.

Due to the applications of the retroreflector mentioned at the beginning of this section, the need for a flat, low-cost, and wideband retroreflector in the microwave frequency range with a wide continuous angular range is strongly felt. To the best of our knowledge, only one retroreflector in microwave domain with non-planar overall structure is presented, which has 3 dB RCS level for the incident angles from − 30° to 30° at 10 GHz and only for TM polarization^14^. In this paper, a novel planar, metal-only, and wideband retroreflector with omnidirectional angular range in microwave frequency is designed. The proposed structure is inspired by the theory of cat's eye retroreflector, which is implemented using transmitarray structures. The transmitarray structure is designed based on the generalized multifocal beam scanning approach in such a way that it focuses the incident wave with different incident angles on a flat plane and the direction of focused waves is almost perpendicular to the flat plane. In this case, by placing a metal plate which coincides with this focused beam plane, the whole structure behaves as an effective planar retroreflector with wide-angle operation range.

## Design procedure

### Theoretical background

**Figure 1:**
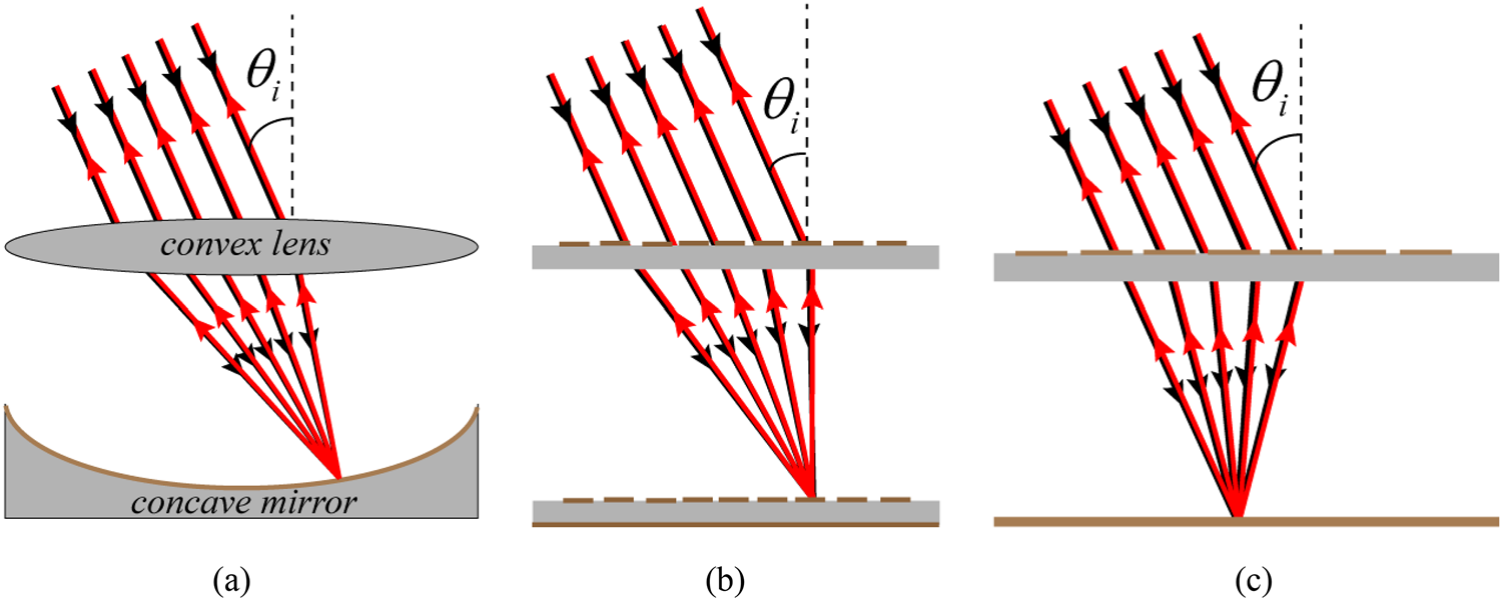
(**a**) Illustration of a conventional cat’s eye retroreflector composed of a convex lens and a concave mirror placed behind the lens. (**b**) Illustration of a planar retroreflector of the first method. (**c**) Illustration of a planar retroreflector of the second method.

The basic theory that this work inspired by it is the theory of cat’s eye retroreflectors. A conventional cat’s eye retroreflector is shown in Fig. 1a that comprises a convex lens and a concave mirror placed behind the lens. As can be seen, the convex lens converges the incident wave with different incident angles to different locations on the concave mirror and since the mirror surface is perpendicular to the direction of the focused waves, the focused waves bounce back along the direction of focus. There are two methods to flatten a cat’s eye retroreflector:

- The structure of the lens is designed to concentrate the incident wave with different incident angles to different locations but on a flat surface, and the reflective structure is a spatially varying phase gradient metasurface, which is placed in the location of the focal plane and is designed in such a way that the focused waves reflect back along the direction of focus. The illustration of this type of planar cat’s eye retroreflector is shown in Fig. 1b.
- The structure of the lens is designed to concentrate the incident wave with different incident angles on a flat surface so that the direction of the focused waves is approximately perpendicular to the focal plane and a flat metal plate is placed in the location of the focal plane. The illustration of this type of planar cat’s eye retroreflector is shown in Fig. 1c.

**Figure 2:**
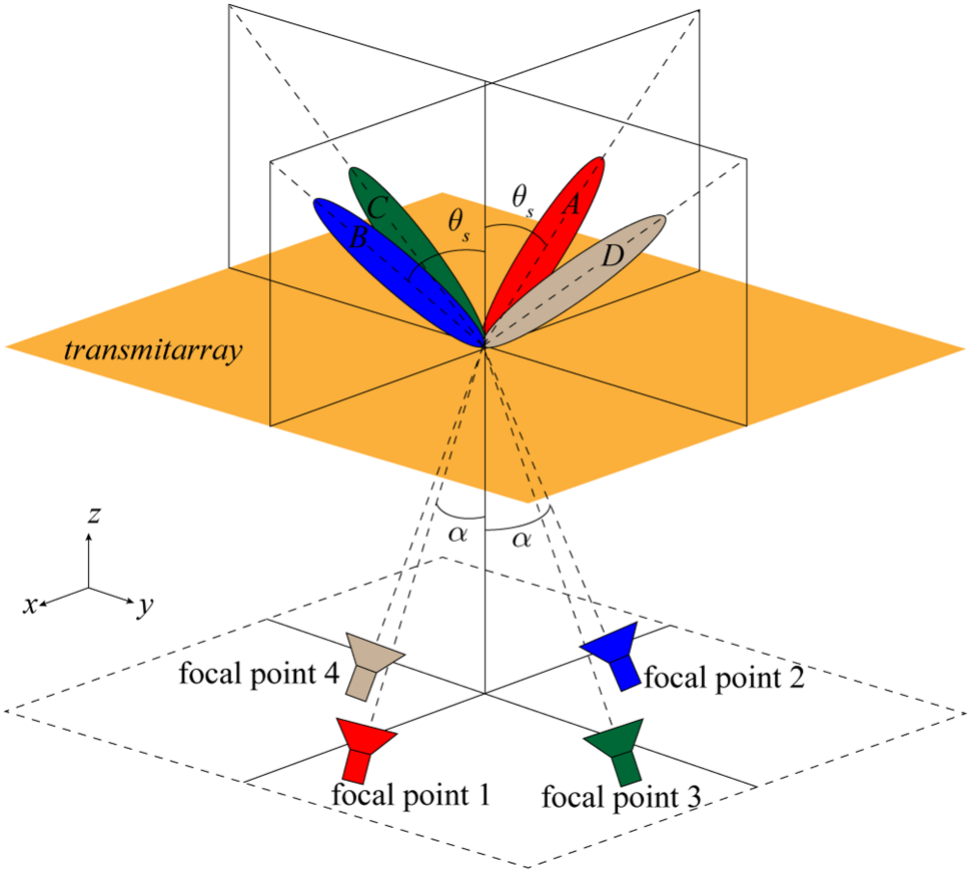
The schematic model of quad focal approach with feed displacement.

In this paper, the second method is followed. The lens structure in cat’s eye retroreflector is implemented with transmitarray structures. Transmitarray antennas are typically composed of one or several focal sources illuminating a planar arrangement of sub-wavelength phase-shifting unit cells designed to define a predetermined phase distribution across the aperture and therefore a predetermined radiation characteristic in far-field or near-field^15^. An approach^16,17^ is proposed for beam scanning called multifocal approach with feed displacement that the quadrufocal case is shown in Fig. 2. This approach says that if the phase of the elements is obtained based on the location of the feed at four focal points and four spatial beams according to Fig. 2, then when the feed in *xz*-plane goes from focal point 1 to focal point 2, the beam moves from mode *A* to mode *B* and it focuses quite well nearby and between the two focal points^15,16^. The same apply to the two focal points in the *yz*-plane. Therefore, according to the reciprocity theorem, planar waves that propagate to the transmitarray antenna at angles $- \theta_{s} < \theta < \theta_{s}$, are focused on a specific curve between points 1 and 2.

### Design of transmitarray structure

**Figure 3:**
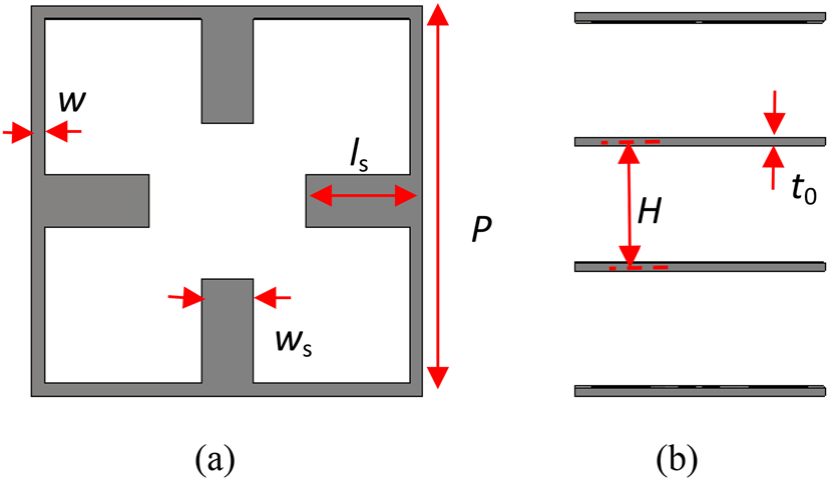
Geometry of the proposed unit cell to support both TE and TM polarization (**a**) top view and (**b**) side view.

The first step in the design of a transmitarray structure is to select the appropriate unit cell. By changing one parameter of the unit cell over a wide frequency band, the transmission magnitude should remain close to 0 dB and the transmission phase should cover from 0 to $2\pi$. In addition, since the full structure with low loss and low cost is required, metal-only unit cells are preferred. In^18^ a wideband metal-only unit cell is proposed that is composed of a square ring within which there are two sets of parallel stubs in front of each other. The square ring and parallel stubs create a notch at zero and a controllable frequency, respectively. Therefore a passband with low slope phase shift response between these two notches is created which leads to a wideband transmitarray. But this unit cell has single linear polarization that makes it unable to support both TE and TM polarizations. Here this unit cell is modified so that four similar stubs are placed within all four sides of the square ring as shown in Fig. 3. This causes the modified unit cell to support both TE and TM polarizations. In this proposed unit cell by adjusting the dimension of the stubs ($l_{p}$), the transmission phase of the unit cell can be controlled. Based on the study of multilayer transmitarrays presented in^19^, a unit cell of four identical layers, separated by quarter-wavelength air gaps, can achieve a full transmission phase range of 360° for transmission magnitudes equal to or better than − 1 dB.

The optimal values of the fixed geometrical parameters are presented in Table 1.

**Table 1:** The optimal dimensions of the proposed unit cell parameters.

Parameter	Value
*w*_s_	2 mm
*w*	0.5 mm
*P*	15 mm
*H*	7.5 mm
*t*_0_	0.5 mm

**Figure 4:**
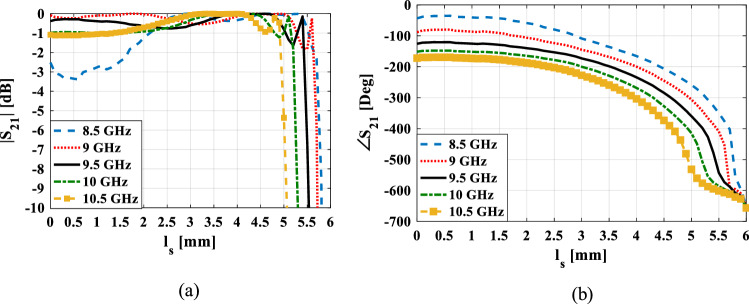
Simulated transmission magnitude (**a**) and phase (**b**) for the four metallic layers unit cell shown in Fig. 3 versus stubs length at five frequencies.

The diagram of the transmission magnitude and phase shift of the unit cell versus different values of $l_{s}$ are shown in Fig. 4 for five different frequencies. It is seen that the phase responses for the five frequencies are almost parallel. Thus, one can expect to have a wideband transmitarray structure. But in the bandwidth estimation, the magnitude response at the five frequencies must also be considered.

**Figure 5:**
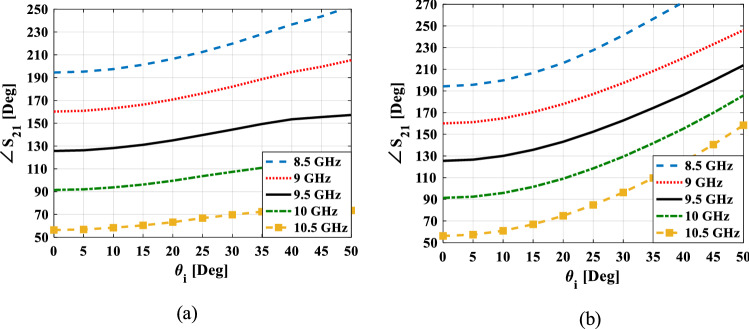
Simulated transmission phase for the unit cell shown in Fig. 3 versus incident angle at five frequencies for (**a**) TE polarization (**b**) TM polarization.

The results shown in Fig. 4 are obtained using the simulation of the unit cell in CST STUDIO software^21^ in which x- and y-boundaries were set to “unit cell” to model an infinite array of that unit cell. In this case, the mutual coupling between unit cells is also included in the simulation results. At the z-boundaries, two floquet ports are set up on both sides of the unit cell and the first two floquet modes that their polarizations are along the y-axis (TE(0.0)) and the x-axis (TM(0,0)) are excited. It is usually assumed that these floquet modes are normally incident on all elements. But in retroreflector structures, the elements are also illuminated by oblique incidence angles. Thus, it is worthy to present the behavior of the proposed unit cell under oblique incidence. Figure 5 depicts the variations in the transmission phase at different oblique incidence angles for TE and TM polarization. From this figure, it can be seen that for TE polarization, the phase error value in 30° incident angle to normal incidence is lower than 18° and for TM polarization is lower than 40° in the whole specified frequency band. Therefore, it is predicted that the structure for TE polarization will provide better results than TM polarization. To moderate the effect of the phase error, the stub length corresponding to the phase of each element is obtained based on the curve of the stub length versus the average of the unit cell transmission phase at the incident angles of 0°and 30°.

The second step in the design of a transmitarray structure is to determine the phase of the elements. As mentioned in the previous section, the phase of the elements is obtained based on the multi-focal approach. It is well known that the elements phase in reflectarray and transmitarray antennas depending on the location of the feed and the direction of the scanned beam is obtained using the following equation^16^:

1 $$ \varphi_{mn} = k_{0} \left[ {R_{mn} - \sin \theta_{0} (x_{mn} \cos \varphi_{0} + y_{mn} \sin \varphi_{0} )} \right] $$

where $\varphi_{mn}$ is the phase of *mn*th element, $R_{mn}$ is the distance between feed and *mn*th element, and $(\theta_{0} ,\varphi_{0} )$ is the direction of scanned beam. Based on the multi-focal approach, the phase of the elements for a symmetric system with four focal points, as in Fig. 2, is obtained as:

2 $$ \varphi_{mn} = \frac{{\varphi_{mn}^{{f_{1} }} + \varphi_{mn}^{{f_{2} }} + \varphi_{mn}^{{f_{3} }} + \varphi_{mn}^{{f_{4} }} }}{4} $$

where $\varphi_{mn}^{{f_{i} }} = k_{0} R_{mn}^{{f_{i} }} , \, i = 1,2,3,4$. Due to the symmetry of the system, the second term in the elements phase equation (Eq. 1) for both focal points facing each other, cancel each other. Therefore the direction of the main beam of the transmitarray will now depend on the feed offset angle, $\alpha$. An important parameter in dealing with beam scanning is the beam deviation factor (BDF) which is defined as $BDF = \theta_{s} /\alpha$, where $\theta_{s}$ and $\alpha$ are the main beam direction and the feed offset angle, respectively. In transmitarray antennas $\theta_{s} = 30^{ \circ }$ and *BDF* = 0.9009 are usually considered^17^.

**Figure 6:**
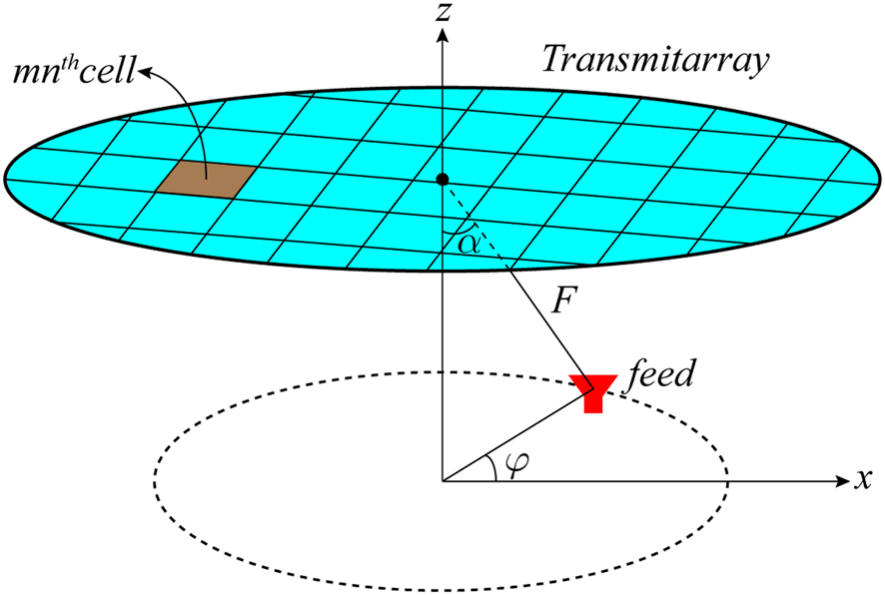
The feed placement locations to achieve retroreflectivity in all azimuth directions.

To have retroreflectivity in all azimuth directions $(\varphi_{i} )$, the transmitarray structure must be symmetrical with respect to $\varphi$.To achieve this, the phase of the elements must be obtained by assuming that the feed moves on a circle according to Fig. 6. Therefore, to calculate the phase of each element, Eq. 2 is extended for all points on the circle, which changes to an integral relation as follows:

3 $$ \phi_{mn} = \frac{{k_{0} }}{2\pi }\int\limits_{0}^{2\pi } {\sqrt {\left[ {F\sin (\alpha )\cos (\varphi ) - x_{mn} } \right]^{2} + \left[ {F\sin (\alpha )\sin (\varphi ) - y_{mn} } \right]^{2} + \left[ {F\cos (\alpha )} \right]^{2} } \, d\varphi } $$

To maintain the overall symmetry of the structure with respect to the azimuth angle, the overall shape of the transmitarray structure is considered as a circle. Here the diameter of the circle is selected $D = 15 \times P$, where *P* is the periodicity of the unit cell.

**Figure 7:**
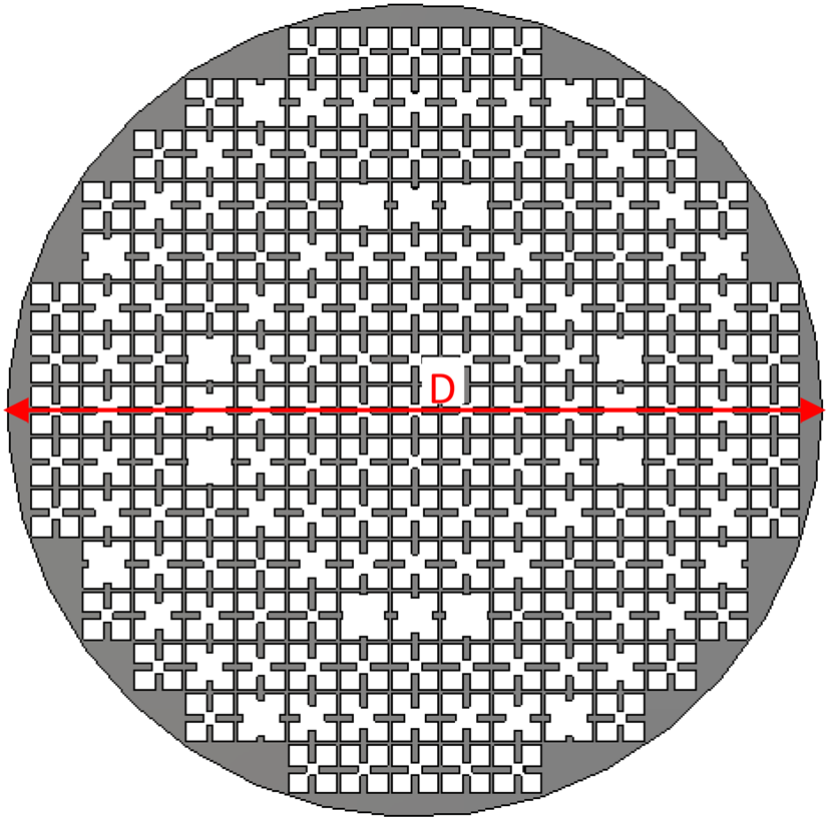
The schematic of the simulated transmitarray structure.

The structure of the transmitarray simulated in CST STUDIO^21^ full-wave software using the unit cell mentioned in Fig. 3 is shown in Fig. 7.

**Figure 8:**
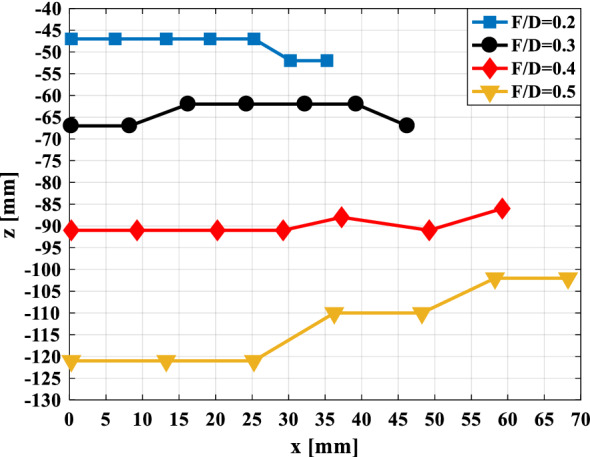
The focused spot locations for four values of $F/D = 0.2,0.3,0.4,0.5$, in different incident angles.

The last step in the design of a transmitarray structure is to select the appropriate *F/D* value. This parameter affects the spatial coordinate of focused spots. Therefore, first, the effect of changing this parameter on the focused spot locations for different incident angles is investigated. To do this, for four values of $F/D = 0.2, \, 0.3, \, 0.4,0.5$, the focused spot locations are plotted for incident angles $\theta_{i} = 0^{ \circ } ,5^{ \circ } ,10^{ \circ } ,15^{ \circ } ,20^{ \circ } ,25^{ \circ } ,30^{ \circ }$ that are shown in Fig. 8. As can be seen, for $F/D \le 0.4$ the location of the focused spots for different incident angles are approximately on a flat surface with a certain *z* value.

Now to more accurately determine the value of *F/D*, the focused beam angle, as well as the amplitude of the electric field at the focused spots, are obtained for different incident angles and for different values of *F/D*. The first one is done by placing a circular metal strip beneath the transmitarray structure at the focused spot location for a specific incident angle. By changing the slope of this metal strip, the angle for which the maximum monostatic RCS is obtained is extracted, which is equal to the angle of focused waves for a certain incident angle. The amplitude of the electric field at the focused spots indicates the ability of the transmitarray structure to concentrate the beam and is obtained by simulating the transmitarray structure alone in CST STUDIO^21^. The values of angle $\alpha$, as well as the amplitude of the electric field at the focused spots for different focused spot locations and different *F/D* values, are given in Table 2. As can be seen, the lower the *F/D*, the smaller the focused waves angle relative to the vertical axis for different incident angles, but also the smaller the amplitude of the electric field at the focused spots for different incident angles.

**Table 2:** The values of angle $\alpha$ as well as the amplitude of the electric field at the focused spots for different focused spot locations and different *F/D* values.

**F/D = 0.2**							
$\theta_{i} \,(^{^\circ } )$	0	5	10	15	20	25	30
$\alpha \,(^{^\circ } )$	0	2	3	3	2	4	5
$E \, ({\text{v/m}})$	5	4.88	4.58	4.24	3.87	3.54	3.27
**F/D = 0.3**							
$\theta_{i} \,(^{^\circ } )$	0	5	10	15	20	25	30
$\alpha \,(^{^\circ } )$	0	0	2	1	0	3	5
$E \, ({\text{v/m}})$	6.11	6.04	5.78	5.42	4.95	4.3	3.94

**Figure 9:**
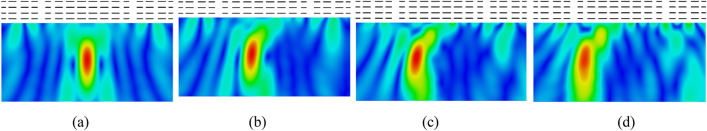
The electric field distribution on x–z plane for the designed transmitarray structure with *F/D* = 0.3 is illuminated by a plane wave with (**a**) $\theta_{i} = 0^{ \circ }$, (**b**) $\theta_{i} = 10^{ \circ }$, (**c**) $\theta_{i} = 20^{ \circ }$, and (**d**) $\theta_{i} = 30^{ \circ }$.

From the above discussions, it is concluded that there should be a compromise between the range of changes of the focused spot location along *z*-axis and the minimum amplitude of the electric field at the focused spots. Therefore, according to Fig. 8, the value of *F/D* is selected 0.3. The electric field distribution on x–z plane for the designed transmitarray structure with *F/D* = 0.3 which is illuminated by a plane wave with different incident angles is shown in Fig. 9. As can be seen, the concentration of the incident wave at one point by the designed transmitarray structure is visible.

## Simulation and fabrication

**Figure 10:**
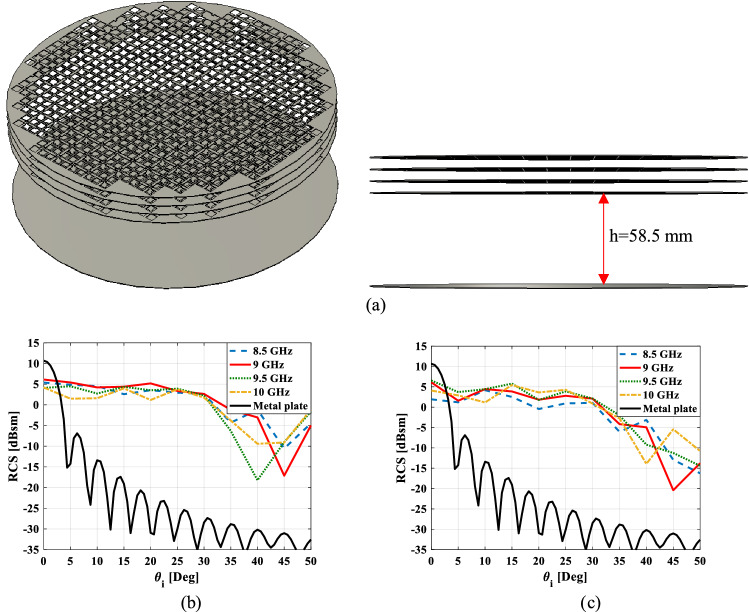
(**a**) The proposed retroreflector structure that is simulated in CST software, the monostatic RCS diagram versus incident angle at four frequencies for (**b**) TE and (**c**) TM polarization.

The proposed retroreflector structure that is simulated in CST software^21^ is shown in Fig. 10a. The simulation results of the monostatic RCS diagram versus incident angle at four frequencies for TE and TM polarization are given in Fig. 10b,c, respectively. As can be seen, the proposed retroreflector can realize the retroreflectivity property with a continuous wide incident angle view from $- 30^{ \circ }$ to $30^{ \circ }$ within a stable 3 dB RCS level in the frequency range of 8.5–10 GHz (approximately 17%) for both TE and TM polarization. In^20^, a quantity is presented to measure the RCS enhancement of a retroreflector. This quantity called $E(\varphi_{i} )$ represents the average of RCS enhancement of the retroreflector relative to a metal plate with the same dimensions on a range of elevation incident angles ($\theta_{i}$) for a specified azimuth incident angle ($\varphi_{i}$). Here, this quantity is obtained E = 27.74 dB and E = 25.45 dB at frequency 9 GHz for TE and TM polarization, respectively. Two points can be extracted from Fig. 10b,c. First, the greater the incident angle, the lower the monostatic RCS (and consequently the retroreflectivity) of the retroreflector. This is because the transmission phase of the unit cell used in the transmitarray section is obtained for normal incidence, resulting in a phase error in oblique incidences. This phase error increases with increasing incident angle. The second point is that the monostatic RCS results for TE polarization are better than TM polarization. This is because, as can be seen in Fig. 5, the phase error of the unit cells in oblique incidence for TE polarization is less than TM polarization.

**Figure 11:**
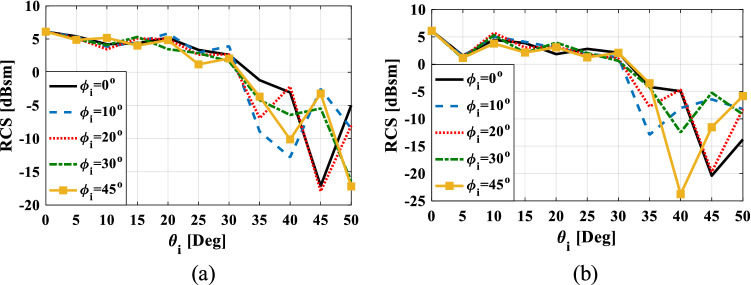
The monostatic RCS diagram of the proposed retroreflector versus elevation incident angle for azimuth angles of $\phi_{i} = 0^{ \circ } ,10^{ \circ } ,20^{ \circ } ,30^{ \circ } ,45^{ \circ }$ at frequency 9 GHz and for (**a**) TE polarization and (**b**) TM polarization.

To verify the omnidirectional property of the proposed retroreflector, the simulation results of the monostatic RCS diagram versus elevation incident angle for five different azimuth angles at frequency 9 GHz and for TE polarization are drawn in Fig. 11. As can be seen, in all five azimuth incident angles $(\varphi_{i} )$ the monostatic RCS diagram is almost the same up to the elevation incident angle of $30^{ \circ }$. Therefore, it can be concluded that the proposed structure has omnidirectional property.

**Figure 12:**
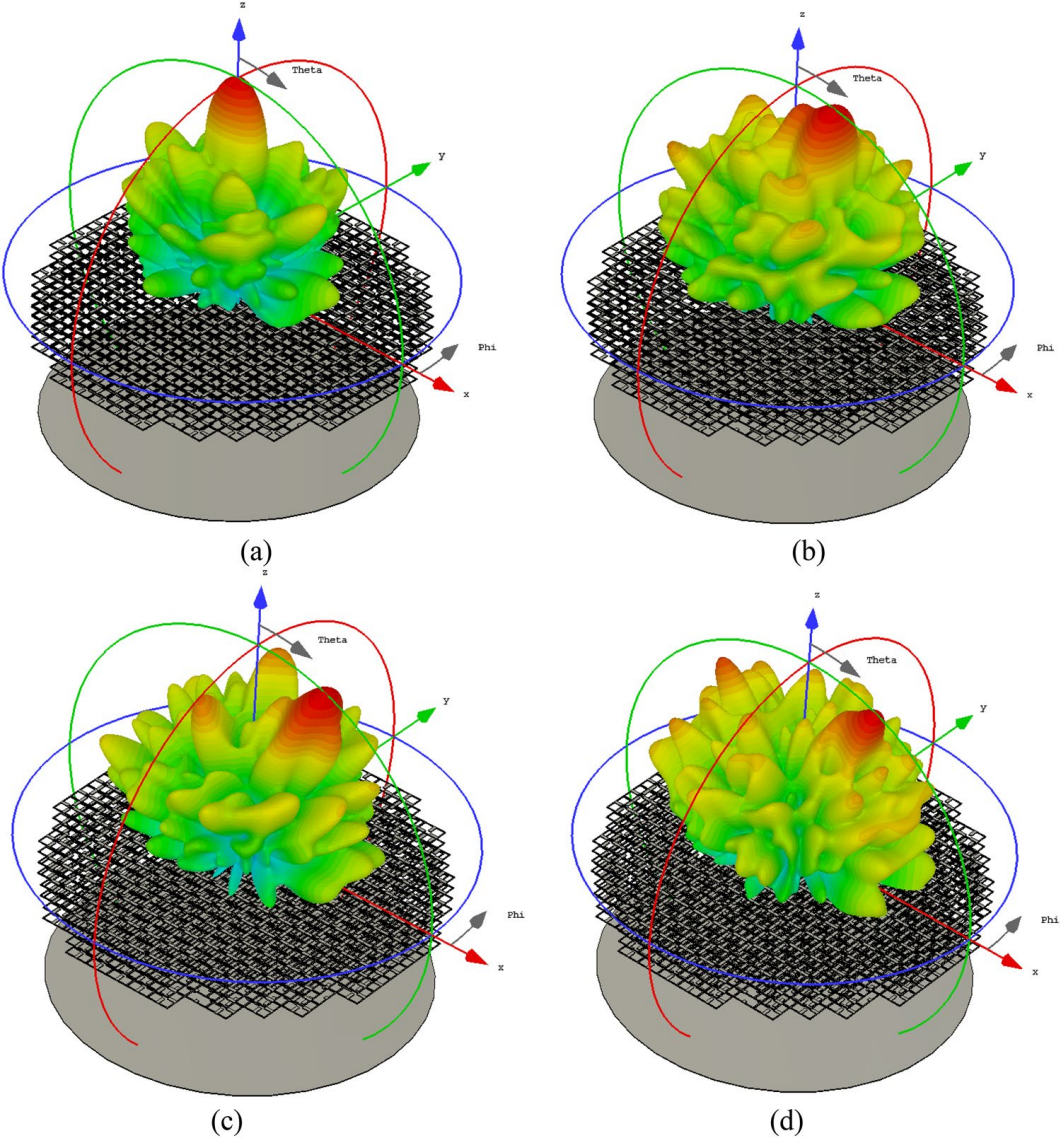
The bi-static RCS pattern for (**a**) $\theta_{i} = 0^{ \circ }$, (**b**) $\theta_{i} = 10^{ \circ }$, (**c**) $\theta_{i} = 20^{ \circ }$ and (**d**) $\theta_{i} = 30^{ \circ }$.

The 3D scattering pattern for the designed retroreflector structure which is illuminated by a plane wave with different incident angles, is shown in Fig. 12. As can be seen, the retroreflector can effectively bounce back the electric field along its incident direction.

**Figure 13:**
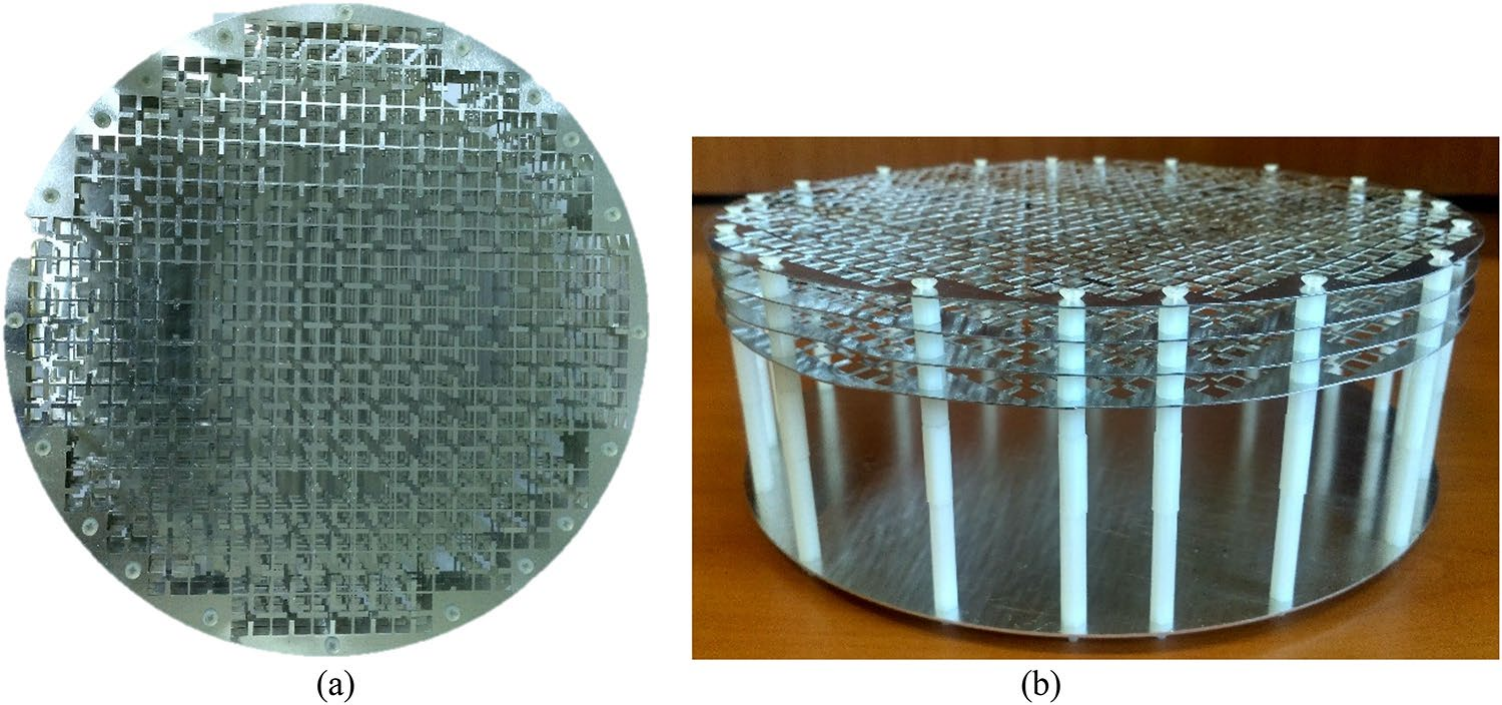
The top (**a**) and side view (**b**) of the fabricated retroreflector.

**Figure 14:**
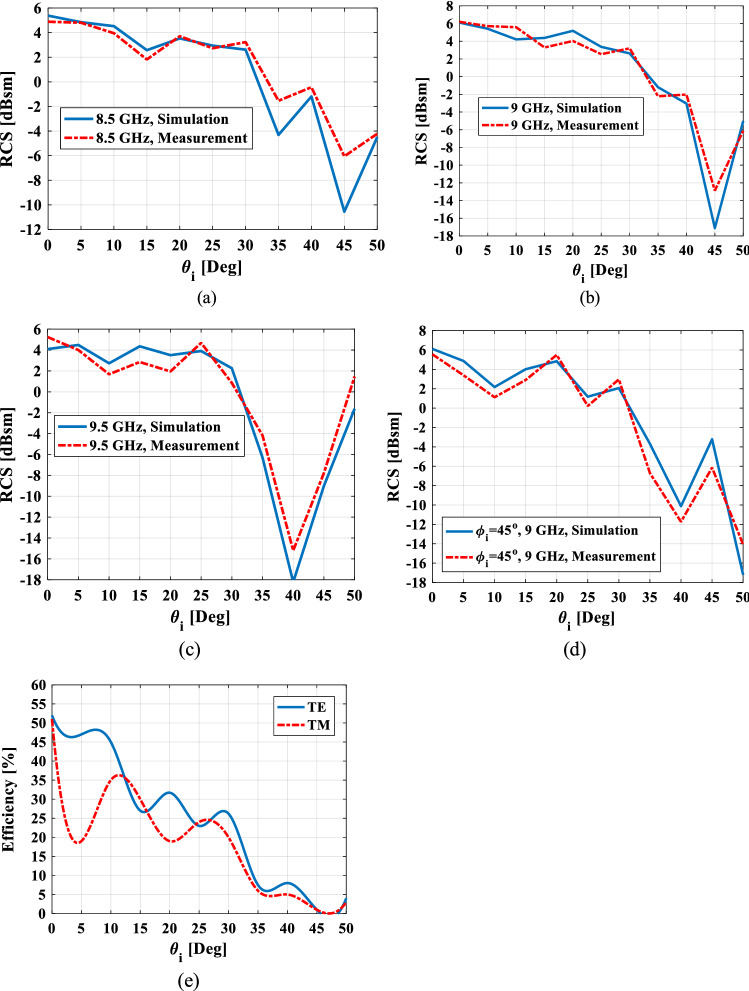
Simulation and measurement results of the proposed retroreflector at (**a**) $\phi_{i} = 0^{ \circ }$, f = 8.5 GHz, (**b**) $\phi_{i} = 0^{ \circ }$, f = 9 GHz, (**c**) $\phi_{i} = 0^{ \circ }$, f = 9.5 GHz, (**d**) $\phi_{i} = 45^{ \circ }$, f = 9 GHz, (e) measured efficiency as a function of incident angle at frequency of 9 GHz for TE and TM polarization.

To validate the proposed retroreflector with simulation results, one prototype is fabricated as shown in Fig. 13. The fabrication is done using laser cutting technology with the fabrication accuracy of $\pm \,0.03\,{\text{mm}}$ and the transmitarray and ground structures are cut on an aluminum sheet with 0.5 mm and 1 mm thickness, respectively. The overall dimension of the retroreflector is a circular shape with a diameter of 225 mm. The measured monostatic RCS of manufactured structure at frequencies of 8.5 GHz, 9 GHz, and 9.5 GHz, as well as the simulation results, are shown in Fig. 14a–d. As can be seen, there is a reasonable agreement between them up to the elevation incident angle of 30°. To calculate the efficiency of the fabricated retroreflector at a certain frequency, first the RCS of an aluminum plate with the same dimensions is obtained for normal incidence at the desired frequency, and it is assumed that this condition is equivalent to 100% efficiency which causes the complete reflection of the incident power. Then the RCS of the fabricated retroreflector for different incident angles at the desired frequency is compared with this RCS and the efficiency of the structure is obtained. The calculated efficiency of the retroreflector versus elevation incident angle at frequency of 9 GHz for both TE and TM polarization is shown in Fig. 14e. As can be seen the minimum efficiency up to incident angle of 30° is obtained 25% for TE polarization and 19% for TM polarization. Also, the results show the half-power (3-dB RCS level) elevation field of view of 60° (− 30° to 30°).

To better determine the characteristics of our work, a comparison between our work and reference^14^ is illustrated in Table 3.

**Table 3:** Comparison of the proposed retroreflector with the reference^14^.

Ref	Half-power (3-dB RCS level) elevation field of view	Omnidirectional property	Half-power bandwidth	F/D	Metal-only	Incident wave polarization
^14^	$60^{ \circ }$	NO	Only one frequency	0.46	NO	Only TM
Our work	$60^{ \circ }$	YES	$\sim 17\%$	0.3	YES	TM and TE

## Conclusions

In this paper, a novel planar and wideband metal-only retroreflector was proposed that covers omnidirectional incident angle range. The proposed structure is inspired by the theory of cat's eye retroreflector in which a symmetrical transmitarray structure with beam scanning capability act as a concave lens and a metal plate act as a mirror. The transmitarray structure is designed based on the generalized multifocal beam scanning approach in such a way that it focuses the incident wave with different incident angles on a flat plane and the direction of focused waves is almost perpendicular to the flat plane. In this case, by placing a metal plate which coincides with this focused beam plane, the whole structure behaves as an effective planar retroreflector with wide-angle operation range. One prototype of the proposed retroreflector was fabricated and tested. The results of measurement show an omnidirectional retroreflectivity with half-power (3-dB RCS level) elevation field of view of 60° (− 30° to 30°) in the frequency range of 8.5–10 GHz (approximately 17%) for both TE and TM polarization.

## Data Availability

The datasets generated during and/or analyzed during the current study are available from the corresponding author on reasonable request.
